# Improving gastrointestinal cancer therapy by uniting stakeholders

**DOI:** 10.1016/j.esmogo.2024.100040

**Published:** 2024-02-15

**Authors:** M. Quante, A. Saborowski, C.B. Westphalen, 2023 Translational and Precision GI-Oncology—Bench to Bedside Meeting

**Affiliations:** 1 Medizinische Klinik II Universitätsklinikum Freiburg, Universität Freiburg, Freiburg; 2 Department of Gastroenterology, Hepatology, Infectious Diseases and Endocrinology, Hannover Medical School, Hannover; 3 Department of Medicine III, University Hospital, LMU Munich, Comprehensive Cancer Center (CCC Munich LMU), German Cancer Consortium (DKTK), Partner Site Munich, Munich, Germany

**Keywords:** precision medicine, GI oncology, academic research, industry participation, technology

## Abstract

Gastrointestinal (GI) cancer—an umbrella term for cancers that affect the digestive system and other abdominal organs—includes some malignancies with the lowest survival rates. To improve patient outcomes, patients must have access to optimal treatment including precision oncology, a method of determining the most effective treatment for an individual cancer patient by identifying predictive biomarkers through the use of advanced molecular diagnostics. While many powerful molecular profiling technologies have already been developed, their uptake in the management of GI cancers could be improved. To bridge this gap, better coordination among three interdependent stakeholders—scientists, clinicians, and industry professionals—is essential. The first Translational and Precision GI-Oncology—Bench to Bedside Meeting was held on 23-28 April 2023 in Freiburg, Germany. The 3-day meeting offered the opportunity for scientists, clinicians, and industry professionals in the field of GI cancer to exchange ideas about how to improve the translation of basic science into clinical practice. One-hundred and twenty participants attended the meeting, which featured 47 presentations covering the following five topics: bedside, analytical approaches for treatment and real-world data, new ideas and biomarkers, novel technologies, and drugs. A summary of the 2023 meeting is provided in this report.

## Highlights

• Gastrointestinal cancer belongs to challenging malignancies with low survival rates.
• Precision oncology using molecular diagnostics is crucial for patient outcomes.
• Molecular profiling technologies in managing these cancers need enhancement.
• Collaboration between academia and industry can advance research and treatment.
• The Translational and Precision GI-Oncology Meeting facilitated exchange of ideas.

## Introduction

Gastrointestinal (GI) cancer—an umbrella term for cancers that affect the digestive system and other abdominal organs—includes some malignancies with the lowest survival rates. To improve patient outcomes, patients must have access to optimal treatment including precision oncology, a method of determining the most effective treatment for an individual cancer patient by identifying predictive biomarkers through the use of advanced molecular diagnostics.^1^ While many powerful molecular profiling technologies have already been developed, their uptake in the management of GI cancers could be improved. To bridge this gap, better coordination among three interdependent stakeholders—scientists, clinicians, and industry professionals—is essential.

The first Translational and Precision GI-Oncology—Bench to Bedside Meeting was held on 23-28 April 2023 in Freiburg, Germany. The 3-day meeting offered the opportunity for scientists, clinicians, and industry professionals in the field of GI cancer to exchange ideas about how to improve the translation of basic science into clinical practice. One-hundred and twenty participants attended the meeting, which featured 47 presentations covering the following five topics: bedside, analytical approaches for treatment and real-world data, new ideas and biomarkers, novel technologies, and drugs. A summary of the 2023 meeting is provided in this report.

## Bedside

Bridging the gap between basic science and clinical medicine, the clinician scientist is a key stakeholder with valuable insight into the scientific and clinical challenges in the treatment of GI cancer. A number of clinician scientists provided an overview of the current standards.

Based on the example of microsatellite-instable cancers, the importance of upcoming immune-oncological treatments of GI cancer was discussed, highlighting a patient-centered multidisciplinary discussion when creating a treatment plan for individuals with metastatic colorectal cancer characterized by microsatellite instability. From a translational point of view, rigorous inclusion of suitable patients into clinical trials is essential in order to better understand prioritization and strategic sequencing of novel therapeutic strategies, including surgical approaches, ‘classical’ chemotherapy, and immunotherapy.^2^

Based on the discussions around the example of microsatellite instability, it is now essential to understand and outline the optimal diagnostic strategy for all patients with GI malignancies to allow for optimal treatment. Thus, the importance of quality-assured molecular testing in a growing number of solid tumors was discussed. It was stressed that this is particularly important because molecular testing serves as the gateway to further treatment decisions, as well as preventive strategies in cancer families. In the future, genomic profiles should increasingly be integrated into the classifications of the malignant disease.

Emerging artificial intelligence (AI) technologies offer the potential to enhance the diagnostic insights available to health care providers and their patients. During the workshop, we discussed the example of label-free digital pathology and its potential implementation into the diagnostic work-up of patients with colorectal cancer. This method utilizes infrared imaging of tissue sections and cellular deposits, combined with advanced AI algorithms, to achieve precise molecular identification of tumors. This AI-assisted technique can help to identify malignancies with a smaller tissue sample size than that required by traditional pathology methods.^3^

While the integration of advanced molecular diagnostics into the management of patients with lung cancer has served as the paradigm for personalized cancer care, patients with GI cancers increasingly benefit from targeted treatment options. Especially in the field of biliary cancer, precision oncology has become the standard of care for a relevant subset of patients. In view of the inevitable development of secondary resistance and considering that only a subset of molecular stratified patients respond to targeted therapies, the key developmental potential of combination therapies in treating biliary cancer was discussed. In particular, the effectiveness of combining active agents—such as two different molecular agents—based on genomic profiling, as well as re-profiling at the time of resistance, was pointed out. Further, in the space of precision oncology in a rare cancer, a close collaboration between industry and academia appears imperative to facilitate reasonable trial design and strengthen translational programs.^4^

Novel targets and therapeutic options need to be further investigated in all types of cancer, and pancreatic cancer in particular is an aggressive malignancy with a high unmet clinical need. The options for targeted therapies are currently limited for patients with *KRAS*-mutant pancreatic cancer. Investigating the effect of transcriptional signatures on treatment outcomes might allow for optimization of treatment algorithms using approved agents. This was highlighted based on a signature predicting sensibility to gemcitabine treatment.^5^^,^^6^

When designing new treatment strategies in cancer, it is critical to recognize the nuances in disease categorization. Current evidence suggests that gastric and esophageal adenocarcinomas, traditionally viewed as separate entities due to different origins, exhibit significant molecular overlap, indicating they may effectively constitute a single disease with implications for unified treatment approaches. However, a clear demarcation remains between adenocarcinoma and squamous cell carcinoma of the esophagus. It is imperative for the pharmaceutical industry to delineate these distinctions accurately to enhance therapeutic strategies and, ultimately, patient survival outcomes.

While significant progress has been made in the treatment of certain subgroups of patients with GI malignancies, a plethora of challenges remain. Outlining examples below shows where concerted efforts and public–private partnerships are needed to improve patients’ outcome.

• *KRAS*-mutant pancreatic cancer
• *KRAS*-mutant colorectal cancer
• BRAF-mutant colorectal cancer
• Right-sided colorectal cancer
• Patients with hepatocellular carcinoma (HCC) and impaired liver function
• Patients with HCC resistant to immunotherapy

## Individualizing patient treatment—practical and analytical considerations

A number of cutting-edge precision medicine approaches show promise in improving patient outcomes when treating various types of GI cancers. Precision oncology in pancreatic ductal adenocarcinoma (PDAC) faces unique challenges due to the high prevalence of RAS mutations, which are found in the majority of PDAC cases and are notoriously difficult to target therapeutically. Despite the promise of precision medicine in other cancer types, PDAC has remained largely intractable to this approach because current targeted therapies are largely ineffective against the majority of RAS-driven tumors. Acknowledging this, there is a potential path forward that involves a two-pronged strategy. The first involves the identification and development of novel biomarkers beyond RAS mutations that could help stratify patients more effectively. For instance, molecular profiling could uncover vulnerabilities in certain subsets of PDAC. Secondly, the integration of ‘co-clinical’ approaches, such as patient-derived organoids, might support in the search for more individualized treatment strategies.^6^^,^^7^

Annotating the tumor immune microenvironment is pivotal in advancing cancer therapeutics. Focusing on HCC, recent advances have been made through the application of imaging mass cytometry (IMC). This technology, which enables high-dimensional cellular analysis and phenotypic profiling, has yielded profound insights into the immune landscape of HCC. IMC facilitates the detailed examination of tumor-infiltrating immune populations and their functional states, contributing to a nuanced understanding of the tumor–immune interplay.^8^^,^^9^

The integration of IMC findings supports novel approaches that aim to further enhance the efficacy of ‘traditional’ immunotherapies for HCC. One such approach targets tumor-associated macrophages to disrupt their support of tumor growth and immune evasion. Concurrently, leveraging checkpoint inhibitors, specifically programmed cell death protein 1 (PD-1)- and cytotoxic T-lymphocyte-associated protein 4 (CTLA-4)-blocking antibodies, could modulate the immune response to enhance antitumor activity.^9^

In parallel, the criticality of precision in the preanalytical phase of cancer diagnostics is underscored. This phase, which spans from the collection of patient biomarkers to data analysis, is notoriously susceptible to variability, thereby affecting diagnostic accuracy and treatment efficacy. Proposals to standardize the preanalytical workflow are essential, encompassing the stabilization of biospecimens during transportation and the meticulous execution of analytical protocols. Such standardization efforts are fundamental to ensuring the reliability of biomarker assessments and, by extension, the customization of patient treatment plans.^5^

A critical avenue of research in oncology is deciphering the mechanisms underlying resistance to targeted therapies. For instance, resistance to BRAF inhibitors in colorectal cancer represents a significant clinical challenge. During the workshop, a study was presented that probes the resistance mechanisms to BRAF-targeted therapies, such as encorafenib combined with the epidermal growth factor receptor antibody cetuximab. This research utilizes patient-derived organoid (PDO) models harboring BRAF mutations from individuals who exhibit varying responses to these therapies.^10^^,^^11^

PDOs are cultivated from both treatment-sensitive and -resistant patients to simulate and study the evolution of resistance. These models enable the observation of real-time responses to treatment and the emergence of resistance. By closely mimicking the patient’s tumor biology, PDOs are instrumental in identifying resistance pathways and testing new drug combinations to overcome them.

The goal of this investigative approach is to unveil novel therapeutic strategies that could preempt or counteract resistance to BRAF-targeted treatments. Ultimately, these insights could lead to the development of more durable and effective treatment protocols for patients with BRAF-mutant colorectal cancer, addressing a key obstacle in the management of this malignancy.

On the question of how to better implement personalized oncology into therapies for GI tumors, the need for closer interaction between academia and pharmaceutical companies was emphasized. Furthermore, the importance of a stronger support for investigator-initiated drug trials and molecular registries and wider access to samples from prospective clinical studies was underlined, and the value of integrating translational scientists in advisory boards was highlighted.

Precision oncology is currently benefiting from the application of AI in many ways. One way in particular is through AI-based identification. Work using artificial neural networks—highly complex models with up to billions of parameters that can understand data that cannot be analyzed by computers running conventional software—was discussed. A few key ways AI techniques can benefit patients with GI cancer including predicting intratumor heterogeneity using AI biomarkers, using deep learning in cancer pathology, and combining many types of information into one multimodal AI biomarker were noted.

There are a number of particularly challenging areas for clinicians; tumor-agnostic therapies are one of them. The fact that developing therapeutics for these agents are difficult because the subgroups tend to be very small and clinical trials and approvals are complex was discussed. The important work being done by the European Society for Medical Oncology, an organization that aims to provide a statistically sound taxonomy to support the development and clinical use of tumor-agnostic therapeutics, was highlighted.

A number of areas in GI cancer treatment would benefit from the involvement by more stakeholders, exemplified by the *In Vitro* Diagnostic Medical Devices Regulation, which came into effect April 2017. To improve problematic regulations such as this one, it was suggested that ideally all stakeholders should be involved in the conception of such regulations and once they are in place, an open dialogue should continue. The application of ineffective one-size-fits-all approaches in the process of implementing novel diagnostic tools appears suboptimal, and effort should be taken to shorten approval timelines.

## New ideas and biomarkers

From rethinking the paradigm that explains how cancers evolve, to using new technology to analyze not just a sample of a tumor but a tumor in its entirety, leading GI cancer researchers presented novel ways to approach treatment that are likely to transform the field.

One subject under investigation is how colorectal cancers evolve. Trevor Graham presented on his work in this area, with the goal of identifying new biomarkers and treatments. He proposed a ‘big bang’ model of colorectal cancer evolutionary development that posits that as soon as cancer is detected, it might already contain everything there is to know about the prognosis of that cancer and its sensitivity to treatment.^12^, ^13^, ^14^

Translating preclinical research into clinical trials and industry poses a number of challenges. By researching the heterogeneity of pancreatic tumors, Axel Behrens hopes to learn how to improve treatment but pointed out that it was insufficient to analyze only a sample of the tumor—instead, he stressed the importance of analyzing a complete tumor in order to understand its complex three-dimensional morphology using his technology, Fast Light-microscopic analysis of Antibody-Stained whole organs (FLASH).^15^

An emerging way to better understand cancer evolution is by using high-throughput genetic screens. One example of how these screens can be used is a discovery Roland Rad’s research group made by carrying out a genome-wide perturbation of the pancreas in mice, that the *Foxp1* gene is both an oncogene and early driver of pancreatic cancer in mice.^16^

Complementing these innovative ideas, 10 young investigators from universities throughout Germany presented their exciting work during 5-minute ‘speed science’ talks. They highlighted promising areas of GI cancer research ranging from how organoid models can be used to study intratumoral heterogeneity in pancreatic ductal adenocarcinoma, to the use of AI for data integration, or the annotation of the molecular mechanisms of amino acid metabolism by which therapeutically relevant vulnerabilities can be uncovered in cancer cells.

## Novel technologies

From patient-derived organoids, to proteomic analysis, to digital twins, there are a number of exciting new technologies that are likely to revolutionize the way GI cancers are treated. The benefits of using patient-derived organoids—miniature three-dimensional organs—to test drug response was discussed. Despite their limitations, these organoids are effective patient-individual models that capture intrapatient tumor heterogeneity and make it possible to predict how that patient will respond to the standard-of-care treatment, as well as detecting possible development of drug resistance.^17^

An example of another effective model—a patient-derived xenograft model—was presented. It was suggested that the most effective way to reproduce an accurate tumor microenvironment, and, in turn, to best reproduce a disease could be to use a hybrid model, rather than using just GEMM, syngeneic, orthotopic, patient-derived xenograft, or orthotopic-patient-derived xenograft models separately.

There are a number of powerful tools that can be used in molecular pathology, including proteomic analysis. It was shown that using liquid chromatography along with a mass spectrometry analysis of formalin-fixed paraffin-embedded (FFPE) tissue has the potential to be used to increase diagnostic precision, refine molecular characterization of tumors, identify new biomarkers, and thus help to improve personalized medicine.^18^

The value of droplet microfluidics, an approach in which millions of stabilized, oil-surrounded droplets serve as miniature test tubes, making it possible to carry out large-scale screens using minimal patient material, was introduced.^19^ Providing existing diagnostic labs with the instruments and test kits—the microfluidic chips and the reagents—would enable them to carry out the screens, using samples from hospitals to produce a report detailing the most effective drugs and a therapy recommendation.

Yet another type of model—digital twins of cancer patients—has a promising future. A digital twin modeling platform in which different types of data—including genomics, transcriptomics, as well as imagining and clinical data—are input, converted into a knowledge graph, and integrated into a network using AI was proposed. This makes it possible to isolate patient-specific measurements in order to create a digital twin of a cancer patient that can be used to produce personalized diagnostics and treatments as well as for precision drug discovery and development.^20^^,^^21^

## Industry perspective—how drugs can be brought into the clinic

Thanks to an abundance of new information, including that provided by clinician scientists through their investigation into novel targets and options in a range of GI cancers, stakeholders in industry are uncovering promising new drugs, bispecific antibodies, and drug sensitivity tests to improve patient outcomes.

Industry partners presented on the problems and needs of the industry. They discussed the need to change the way clinical trials are carried out in order to implement novel therapeutic targets. An evidence-based model to improve cancer treatment, which faces challenges throughout the process, from diagnosis, to care plan, to access and treatment, and outcomes and monitoring, was mentioned. Meeting these challenges through accelerating diagnoses, integrating and automating workflows, optimizing treatment planning, and collaborating with partners to utilize state-of-the-art technologies were discussed. A number of key industry partners (Amgen, MSD, Curex, AstraZeneca, Pierre Fabre, PreAnalytiX, Servier, Roche, Novartis, Astellas, Bristol Myers Squibb, Dr. Falk, Lilly, Bayer, Zeiss, Daiichi-Sankyo) made an important contribution to how industry can interact with clinician scientists to improve novel therapeutic strategies and phase I clinical trials by introducing their pipelines.

There are many innovative therapeutics in the pipeline that could help patients with various types of GI cancers. We discussed prevalent targetable alterations in patients with GI cancers, and the potential value of combination strategies, encompassing a selected combination of targeted medication with immune checkpoint inhibitors. We see this pairing as a promising way forward in order to achieving a tumor response while overcoming drug resistance. Further, several bispecific antibodies—for instance, antibodies binding to PD-1 and CTLA-4 that increase internalization and lead to degradation of PD-1—are in clinical development and may help to improve long-term survival in patients with advanced solid tumors.

## Conclusion

The seamless integration of academia and industry is essential for propelling GI cancer research and treatment toward new horizons. To truly harness the strengths of both sectors, fostering a symbiotic collaboration is paramount.

The meeting highlighted several actionable points to enhance this relationship. Our discussions emphasized the profound potential of melding the technological strengths and deep molecular insights of academia with drug development pipelines through collaborative initiatives. This vision extends to the development of shared knowledge exchange dedicated entirely to cutting-edge GI cancer research. A symbiotic relationship can be nurtured by exchange programs that promote the sharing of knowledge. Joint workshops can serve as platforms for leaders from both areas to strategize and ideate.

In addition, clinical trials offer another avenue for collaboration by expediting patient access to innovative molecular treatments. With the guidance of industry experts, the labyrinth of regulatory pathways can be more easily traversed, ensuring a smoother transition of treatments from the lab bench to the bedside. We welcome further unified efforts reflecting the rigor of academic methodologies complemented by the industry’s practical applications. Feedback mechanisms and structured platforms can serve to refine academic ideas, ensuring they meet real-world requirements. Lastly, streamlined licensing processes can ensure that innovations transition swiftly from academia to the market, which would be beneficial for both sectors.

**Figure 1:**
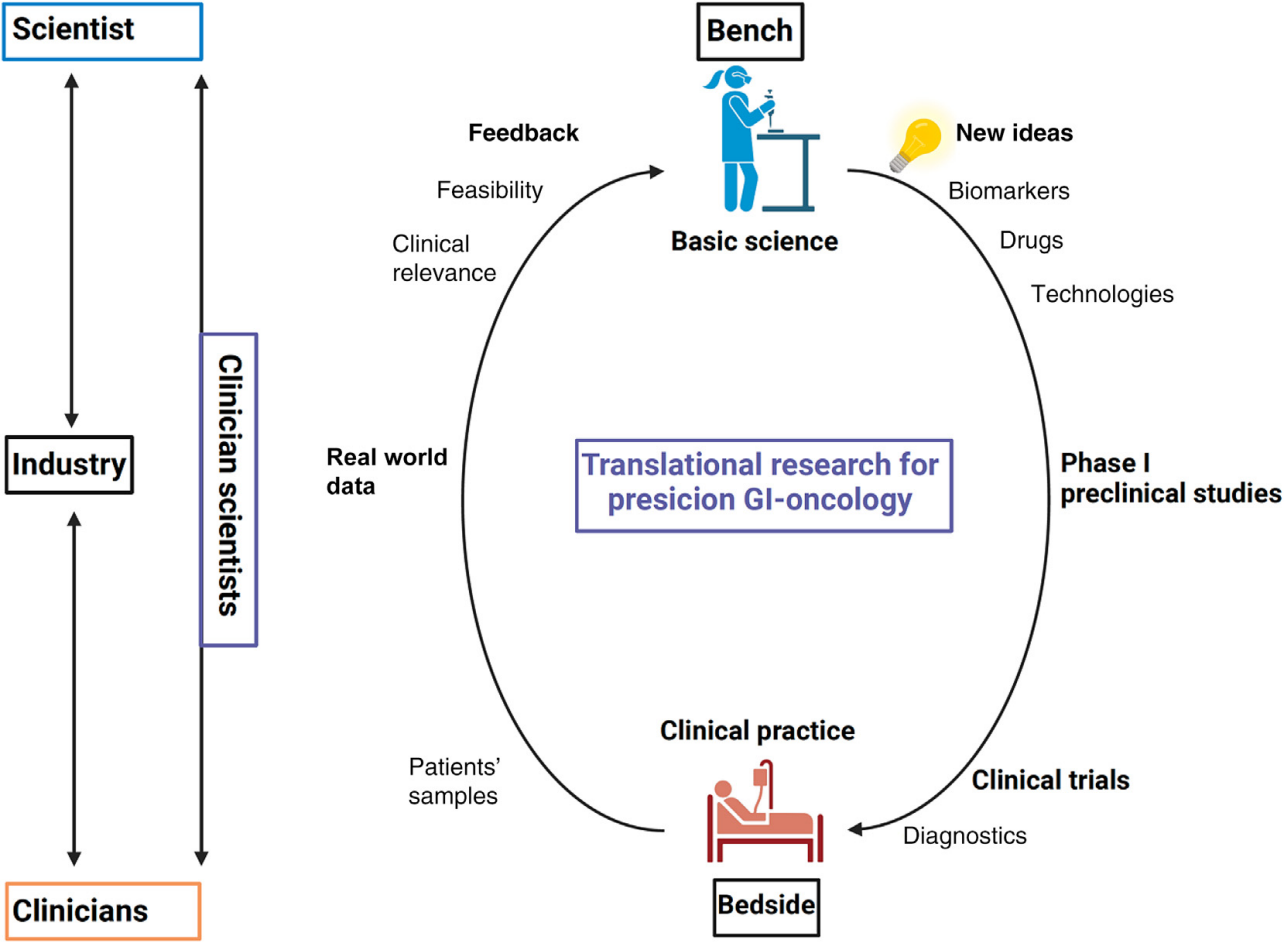
Summarizing a concept of uniting stakeholders and translational research in gastrointestinal oncology (designed by Biorender).

In conclusion, the synergistic union of academia and industry can significantly sculpt the future of GI cancer care. By ardently pursuing these avenues of collaboration, we anticipate a transformation in drug and technology development, bringing hope and healing to patients globally (Figure 1).
