# Supplementary material for: Improving gastrointestinal cancer therapy by uniting stakeholders
Source: ESMO Gastrointest Oncol. 2024 Feb 15;3:100040. doi: 10.1016/j.esmogo.2024.100040 (PMC12836728; doi:10.1016/j.esmogo.2024.100040)
Supplement: Supplementary data [file mmc1.docx]

**Supplement: Meeting Agenda:**

Workshop 1: Need for precision medicine

Overview: Role of Precision Oncology in GI Oncology (B Westphalen, LMU München)

The Molecular Tumorboard and its impact on Clinical Oncology (H. Becker , Freibrug)

Advanced molecular diagnostics: View from Industry perspective (M. Thomas, Roche)

Advanced molecular diagnostics: View of the Pathologist (A. Stenzinger, Heidelberg)

Advanced molecular diagnostics: View of the Bioinformatician (M. Börries, Freiburg)

Workshop 2: Implementation of routine molecular diagnostics – Use Case: MSI

Re-Thinking immune-oncological treatment in Gastrointestinal Cancer (F Pietrantonio, Milano)

What is the best diagnostic method (M Kloor, Heidelberg)

Will Artificial Intelligence help pathology? (K. Gerwert, Bochum)

Best Diagnostic Sequence – Who is responsible for testing? (R. Hüneburg, Bonn)

Keynote:

Cancer evolution and its impact for cancer therapy, T Graham (The Institute of Cancer Research, London)

Speed Science Session 1

Treatment of KRAS mutated pancreatic cancer, the role of the phosphatase SHP2 (K. Frank, TU Munich)

Farewell to ampullary cancer; mixed type (K Al Halabi, Heidelberg)

Pathways shaping PDAC Subtype Identity through Chromatin Remodeling (L Klein, Göttingen)

Microbiome, therapeutic response and subtypes in PDAC (Ch. Ammer-Herrmenau, Göttingen)

PDAC organoid models to study intratumoral heterogeneity (A Papargyriou, TUM)

Day 2:

Keynote:

Translation of preclinical research into clinical trials and industry A. Behrens (Imperial College London)

Workshop 3: Novel Technologies – Ready for clinical implementation?

Organoid models (T Schmäche, Dresden)

Mouse and PDX Models (Shiv K. Singh, Göttingen)

Proteomic analysis (S Singer, Tübingen)

Droplet microfluidics in personalized cancer therapy (Ch. Merten, EPFL Lausanne)

Mathematical models (W. Kolch, University College Dublin)

Workshop 4: New Kids on the Block in GI-Oncology – Industry

Real-world experience with a functional drug sensitivity test (R. Gruber, 2cureX)

Targeting IDH1 in cholangiocarcinoma (T Reisländer, Servier)

Future Pipeline of MSD in GI Oncology (D. Brockelt, MSD)

Bi-specific Antibody treatment in GI oncology (S Dovedi, AstraZeneca)

Targeted therapies for GI tumors from the Amgen pipeline (D Hecker, Amgen)

Workshop 5: New Kids on the Block in GI-Oncology – Academia

Novel targets and options in biliary cancer (A. Saborowski, Hannover)

Novel targets and options in pancreatic cancer (L. Perkhofer, Ulm)

Novel targets and options in gastroesophageal cancer (M. Quante, Freiburg)

Novel targets and options in colorectal cancer (A. Kurreck, Charite)

Novel targets and options in hepatocellular cancer (U Ehmer, TU München)

Speed Science Session 2

1. Myc-dependency of gastric neuroendocrine carcinomas (S Lange, TU München)

2. Kinase inhibitor responses in patient-derived organoids (J Traichel, Freiburg)

3. Multimodal data integration using AI (M Unger, Dresden)

4. Molecular mechanisms of amino acid metabolism in cancer cells (L Hinze, Hannover)

5. Cancer Prevention in gastroesophageal carcinogenesis utilizing FXR agonists (A. Proano-Vasco, Freiburg)

Day 3:

Workshop 6: Promises and Caveats in Precision-Oncology

Biomarker driven precision oncology in PDAC (G. O’Kane, Dublin)

Car-T cell Therapy for GI tumors dream or reality? (S. Kobold, LMU München)

Imaging mass cytometry: Lessons from the cancer microenvironment? (B. Bengsch, Freiburg)

Pre-diagnostic approaches to enable molecular analysis (Uwe Oelmueller, Qiagen/PreAnalytiX)

Resistance mechanisms to BRAF targeted therapy and ways to overcome them (S. Stintzing, Berlin, R. Fritsch, Zürich, Pierre Fabre)

Workshop 7: Implementation of personalized oncology in GI tumor therapy

Wish list of academia for implementing personalized medicine (A. Vogel, Hannover)

AI in precision oncology of GI cancer (N. Kather, Dresden)

Clinical challenges of tumor agnostic therapies (B Westphalen, LMU)

Regulatory aspects for clinical implementation of novel diagnostic tools (T. Bartl, CE plus)

Keynote:

High throughput drug screens (R. Rad, TU München)
